# Hysterical Monomania

**Published:** 1854-03

**Authors:** I. P. Coleman

**Affiliations:** Pemberton


Hysterical Monomania.—By I. P. Coleman, M. D.—In October, 1852, I was called to Mary ——, a young Irish woman; aged 18, affected with what I supposed to be hysterical convulsions. The symptoms were strong muscular movements in both upper and lower extremities; violent contortions of the body, and twistings from side to side, head thrown back, face flushed, eyelids closed and tremulous, arms forcibly brought against the breast, and hands grasping at the throat, with a whining cry of distress. The attendance of two persons was required to keep her in bed.

The use of valerian and cathartics calmed the nervous tumult so satisfactorily that I made but three visits, and heard no more of the case until the following June. At that time rumor was busy upon the strange phenomena taking place at the house of her employer. I went thither, and obtained the following history, viz: the nervous irregularity had been comparatively quiet for some time after my former attendance, but in two or three months increased in frequency and violence, until the paroxysms were frightful, lasting two or three hours, with loss of consiousness, and disturbance of the affective faculties—patient extremely irritable on recovery, assailing the spectators with any missile at hand.—Complained of a knawing and tearing sensation in the stomach, and during the paroxysms the epigastric distention was so great as to excite uncharitable suspicions upon her reputation. Loss of appetite for ordinary food, but drank large quantities of milk when unobserved, as that gave most relief to the internal knawings, and quieted the commotion so frequently present; embonpoint well preserved, and complexion sanguine. The attacks were almost daily, and most frequently in the evening. The girl was fully of the opinion that she contained a living animal in the stomach. Early in June her case was stated to a celebrated doctress, who pronounced it to be either a snake or a tape-worm, and ordered fern-root to be taken in decoction. Apparently much against her inclination, she was induced to drink it, when a dreadful scene ensued. The "*baste*," as she termed it, was offended at the potion, turned his back upon it in a rage, and made desperate lunges to escape through the œsophagus, and once blew sand into her mouth, which she ejected into a basin. The intergastric evolutions continued thirty-six hours, during which time violent spasms an maniacal ravings were almost constant; a calm then ensued, and there was tranquility for four or five days, at the end of which time a new phase was presented. Horrible sensations of strangulation, and violent, but ineffectual efforts to vomit came on, the hands were thrust furiously into the fauces, and fragments of dark tough skin forcibly extracted. The apparent agony during the efforts, was great, each paroxysm terminating with violent hysterical convulsions, turgid face, protruding eyes, throbbing carotids, and bursting jugulars, with seeming unconsciousness. Day after day such things were exhibited, but chiefly in the evening, and always in the presence of several attendants and spectators. The quality of the material changed from skin, apparently having scales, to a soft, solid substance, which when dry, resembled sweet potato in fracture and odor: also, a substance similar to wood having undergone eremacausis, and smelling like mushrooms; also, the green and ferruginous sand of the locality. The above substance to the amount of two or three pints, was forcibly thrown from the throat. then followed viscera, intestines in fragments, making five feet, a small heart, liver and trachea, all free from putrefaction or digestive erosion. At this stage of the proceedings, I first saw the patient, when she was beginning to produce bones, a few joints of small vertebræ resembling the caudal extremity of an eel, ribs, with processes for vertebral articulation, and osseous union to the sternum, with cartilaginous joint in the centre; also, several pieces of dorsal vertebræ, one having a lumbar and sacral termination, and some small bones resembling reptilian extremities. The vertebræ amounted to several inches, and were in sections of two, three, and four joints, united by some muscular and fibrous tissue, bearing the appearance of having been macerated or partially digested. As these specimens were produced at intervals of two or three days, and twelve or fourteen days had elapsed since the first exhibitions of skin, the latter productions evinced more and more the effect of maceration, until the last were nearly denuded. The absence of sound information in comparative anatomy and reptilian habitudes, together with the patient's history, and the testimony of the family,—unimpeachable in veracity, and incapable of collusion,—gave plausibility to opinions which were subsequently disproved. An Irish girl having been in the habit of drinking from a running stream at home, becomes sea-sick on the passage, feels an epigastric gnawing—one month after her arrival has hysterical convulsions, her first derangement in health; gastric irritation increases; reflexed movements in the voluntary muscles cruelly augmented; firm conviction of the existence of a living animal within her, she takes the decoction of fern-root; convulsions aggravated; the animal dies; in six days she begins to eject skin, flesh, viscera, and bones, the first not acted on, the last very much so by the gastric fluids. A strong coincidence in chronological and physiological appearance. Although Ireland is destitute of reptiles in general, the good old Saint has suffered one variety of water lizard to remain, familiarly known as the man-keeper, which tradition says is frequently swallowed. When dissatisfied with his situation, and the unlucky wight also with his tenant, the latter has but to lie down with his mouth near a babbling stream and the reptile makes his exodus. The text books, after enumerating the parasites most common to man, have a stereotyped appendix of accidental occupants—larvæ of flies, triton palustris, lacesta aquatica, salamander, &c., and these are so modified by their new situation, as scarcely to be recognized.

The lacesta aquataca, like the tadpole, for a period after his escape from the ovum, is truly acquatic. He may possibly be swallowed in this stage of existence. Vitality, and the active principle of development in early life were supposed to be capable of resisting the digestive process. His small pulmonic, when compared with the systemic artery, proves him to require a very limited quantity of air for respiration, even less than the fishes. The law of adaptation acting so extensively on inferior animals might so have changed his habitudes as to enable him to abstract a sufficiency of air from the food and saliva. A constantly elevated temperature, and abundance of food, might have increased his development even to monstrosity. But the climax exposed the deception by producing four entire sections of lumbar and sacral vertebræ, also the skeleton and part of the carapace of the land turtle. The artfulness of these transactions adds another example to the catalogue of deceptions practiced by the hysterical, both upon themselves and their friends. I had been present during a number of the paroxysms, had seen her struggles while extracting the fragments of bone from the throat; the hemorrhage from the fauces, and the concluding convulsions, without detecting the trick, although suspecting the possibility from the first, and advising the family to be on their guard for deception. The amount of vertebræ thus exhibited is one foot in length. As there is much vagueness in the minds of physicians as to the kinds of parasites capable of existing in living animals, I give the remarks of Professor Leidy on the subject, who has kindly offered his opinion on this occasion. He says, "the bones consist of six vertebral columns of young pigeons, fragments of cervical vertebræ of the common fowl, and bones of the extremities and head, with fragments of the carapace of a turtle. The heart and intestines appear to be those of a bird. If the collection were vomited from the stomach, it of course must have been previously swallowed. Independent of the character of the animals to which the different parts in the collection belonged, *no animal possessing an osseous skeleton* is capable of living within the *human stomach.* Serpents, lizards, toads, and salamanders, if swallowed alive, most probably would not remain in that condition a single hour; indeed, no animal whatever can live within the human alimentary canal except intestinal worms, and the larvæ of several genera of flies, as the besties or bot-fly, musca or flesh-fly, &c. There have been numerous instances recorded of the supposed existence of eels, serpents, frogs, toads, salamanders, snails, &c., in the human stomach, but upon examination they have invariably proved to be false."

Here is a case of reasoning monomania: the cause of the disease is obscure, as the functions of organic life appeared to well performed, but its character is unmistakable. The contortions described above, distention by flatus, globus hystericus, gastric pains referred to the side described as pathognomonic of hysteria. Predisposed to superstition and the marvelous, it is not singular that the idea of a living animal should be entertained. Esquirol says, "a weak understanding, but little or badly cultivated, pre-disposes to monomania." The absorbing idea in this case, was the reptile, a public declaration of its existence had been made, and an honorable obligation required its demonstration. Cunning, so vivid in the insane, devised the plan, and reason executed it as by inspiration. An ignorant girl, not apparently designing in other respects, conducts the scheme with the accuracy of a physiologist, for a time; thus the nervous agitation increases in force and frequency for several months—she takes medicine; the paroxysms are exalted; in thirty-six hours the commotion subsides; teguments are extracted from the throat, apparently flesh, viscera, bones, at first covered with tissue, gradually less and less so, until they are entirely denuded. Here the judgment failed; too great desire for notoriety exposed the trick, but not the manner of it. Quadruplicates of entire sacral vertebræ, with the skeleton of a turtle, were too much for the largest credulity.

That much of this material came from the stomach, I have no doubt, judging from personal observation and the testimony of witnesses of undoubted sincerity. The ability in some to regurgitate, is great, especially when the fauces are titilated. The absurdities of hysterical whims are proverbial. When delicately educated females drink their own urine, and vomit up to convey the impression that renal function is suspended, and the stomach doing vicarious service; or when the urethra and vagina are filled with pebbles to cheat the doctor, and the hoax not confessed until placed on the table, before a class of students, and tied, as for lithotomy, we are not astonished at any report of extravagance.

Esquirol, again, says, "when monomania does not become chronic it frequently terminates spontaneously, with or without sensible crisis." The girl is now well, and unless the violent revulsions so frequently excited in the stomach at these exhibitions, modified some existing morbid condition of that organ, the cause of recovery is not apparent. Milk is no longer relished, and the appetite is good for ordinary food. As this case excited much interest in the country, and even among the savans of the city, it is offered to give publicity to the declaration of Prof. Leidy, "that no animal possessing an osseous skeleton, is capable of living within the human stomach."

PEMBERTON, *Jan.* 16, 1854.

